# Trends in the utilization of youth primary healthcare services and psychological distress

**DOI:** 10.1186/s12913-021-06124-w

**Published:** 2021-02-03

**Authors:** Thomas Potrebny, Nora Wiium, Anne Haugstvedt, Ragnhild Sollesnes, Bente Wold, Frode Thuen

**Affiliations:** 1 Centre for Evidence-Based Practice, Faculty of Health and Social Sciences, Western Norway University of Applied Sciences, Bergen, Norway; 2 Department of Health Promotion and Development, Faculty of Psychology, University of Bergen, Bergen, Norway; 3 Department of Psychosocial Science, Faculty of Psychology, University of Bergen, Bergen, Norway; 4 Department of Health and Caring Sciences, Faculty of Health and Social Sciences, Western Norway University of Applied Sciences, Bergen, Norway

**Keywords:** Psychological distress, Psychological health complaints, Depressive symptoms, Young people, Youth, Primary healthcare, Service use, Trend

## Abstract

**Background:**

Psychological distress among young people is increasing in Northern Europe. According to established healthcare utilization theory, this will create a greater need for youth primary healthcare and subsequently lead to more help-seeking behavior by distressed young people. The aim of this study was to investigate the association between the use of youth primary healthcare services and psychological distress in times of increasing mental health problems and increased service need.

**Methods:**

This study consisted of five waves of repeated annual cross-sectional data collected from young people (aged 13–19) living in Norway between 2014 and 2018 (*n* = 368,579). Population-weighted and design-adjusted generalized linear regression with a log-link was used to examine the use of youth primary healthcare services over time.

**Results:**

We found that a large proportion of young people use primary healthcare services and that young people with high levels of psychological distress use primary healthcare services twice as much as their peers with low levels of psychological distress. In addition, between 2014 and 2018 both psychological distress and primary healthcare service utilization increased: psychological distress increased by 5% and total primary healthcare service use increased by 500 consultations per 1000 young people. Overall, psychological distress had a conditional association with youth primary healthcare service use and could account for between 16 and 66% of the change in the use of services between 2014 and 2018, depending on the service type. However, the absolute increase seen in the use of primary healthcare services was mainly driven by young people with low levels of psychological distress as opposed to young people with high psychological distress. This suggest a converging trend.

**Conclusions:**

Our findings suggest that there might be serious barriers between need and help-seeking behavior for young people with high levels of psychological distress and that the pattern of utilization among young people with lower distress may indicate overuse, possibly as an inadvertent consequence of a newly introduced school absence policy. While further research is needed to confirm these findings, our work may inform healthcare providers and policy makers about primary healthcare utilization trends among young people.

**Supplementary Information:**

The online version contains supplementary material available at 10.1186/s12913-021-06124-w.

## Background

Indicators of psychological distress among young people, such as psychological and psychosomatic health complaints, appear to be increasing in many developed and Western countries [1–3]. In comparative studies in Europe and North America, young people in the Nordic countries stand out in regard to psychosomatic health complaints where an increasing trend has been reported over the last 20 years [1, 2]. One comparative study highlights Norway as the country with the largest increase in youth reports of two or more weekly psychosomatic complaints, with an increase of almost 11% (from 21.8 to 32.5%) from 1994 to 2010 [2]. Based on the available population health data in Norway, there is further evidence for a trend of increasing psychological distress among young people between 1992 and 2018, especially among females [4–8]. In contrast, the number of health complaints observed in other geographical regions remained stable or declined [1, 2].

Like other countries with an increasing trend of psychological distress among young people, Norway has also seen a parallel increase in the diagnosis of mental illness, prescription of antidepressant medication and primary healthcare service use. These changes appear to correspond with the increase in psychological distress – particularly pronounced among young females [6, 8, 9]. Consequently, deteriorating mental health among young people is recognized as a public health concern both internationally and in Norway [10, 11], and further research to improve our understanding of psychological distress and help-seeking behavior is needed.

Psychological distress is associated with healthcare service use and help-seeking behavior [12, 13]. The use of mental health services by young people is increasing in many developed countries [13–15]. By their own account, mental health problems are among the most common reasons why young people seek help in the primary healthcare service [16]. Alarmingly, young people frequently show the worst service access compared to other age groups [17] even though 75% of all mental disorders emerge before the age of 25 [18]. Another review has shown that only 20–40% of young people with mental health problems are detected by primary healthcare services and only about 25% of the young people who need professional treatment receive it [19]. Homlong et al. [20] found that among 15 to 16-year-olds, frequent users of school health services or youth health centers had a greater risk of dropping out of upper secondary school. A Norwegian report found that one in five upper secondary school dropouts reported that they quit school because of mental health problems [21].

Norway represents a suitable research setting to further explore the association between primary healthcare service utilization and psychological distress among young people, since both psychological distress and service use appear to be increasing.

In Norway, the school health service and youth health centers are statutory primary healthcare services provided specifically to all young people, without having to pay. The Norwegian municipalities organize the activities in these services according to their local needs. These youth-friendly services aim to increase wellbeing, prevent early mortality and morbidity, promote sexual health, reduce mental health issues and reduce inequalities in health. The statutory services provide health checks, counselling, and referrals. They are low-threshold and easily accessible to the public. The school health service is available at all primary, lower secondary and upper secondary schools during school-hours while the youth health centers are available in the community outside school-hours. The statutory services include school nurses, doctors, psychologists and physiotherapists, as well as other healthcare professionals [22].

Beside the primary healthcare service, the specialist healthcare service in Norway include the Division of Children and Adolescents’ Psychiatric Polyclinic Services (BUP), at the next level of care. BUP help children and young people with treatment of more severe mental health issues. This service normally requires a referral through a family doctor (within primary healthcare). When BUP becomes involved this specialist healthcare service collaborates with the youth primary healthcare services [23]. According to the Norwegian institute of public health many cases of mental illness are treated outside of the specialist health service which emphasizes that in the Norwegian healthcare system, youth primary healthcare services are important in preventing mental health problems [24].

The Andersen healthcare utilization model [25] is a widely accepted conceptual model for the study of health services utilization. The model suggests that help-seeking behavior is a complex interaction between three sets of determinants: predisposing factors (demographic and social), enabling factors (economic) and need for care (health outcomes). Based on this theoretical framework, it could be hypothesized that increasing psychological distress among young people will be associated with an increased need for primary healthcare services, such as “youth specific” services that promote health (e.g., youth health centers and school health services) and traditional primary healthcare services (e.g., family doctor, psychologist and out-of-hours primary healthcare). Increased need for care should hold true when considering predisposing factors (age, gender) and enabling factors (socioeconomic status, service availability, ease of access).

The aim of this study is to investigate the relationship between youth primary healthcare service use and psychological distress in times of increasing mental health problems and increased service need. The following research questions will be addressed:

RQ 1: To what extent does psychological distress increase among young people between 2014 and 2018?

RQ 2: To what extent is psychological distress associated with youth primary healthcare service use?

- Can changes in psychological distress explain changes in service use among young people over time?
- How much of the trend in primary healthcare service use can be explained by changes in psychological distress?
- Is there an increasing trend of primary healthcare service use among youth with high levels of psychological distress compared to youth with low levels of distress?

## Methods

### Study design and participants

The data analyzed are drawn from the Norwegian Ungdata (Youngdata in English) national survey. Ungdata is an annually repeated cross-sectional data collection scheme, designed to conduct youth surveys at the municipal level in Norway (for more information see http://www.ungdata.no/English). Ungdata collects the same information from a different sample of individuals each year, allowing samples to be compared over time. A repeated cross-sectional study is an appropriate design when investigating social change and group change over time [26].

Since its inception in 2010, youth surveys have been conducted repeatedly in nearly all Norwegian municipalities. Ungdata is regarded as the most comprehensive source of data on adolescent health and well-being in Norway. The data are used in municipal planning and developmental work related to public health and preventive measures as part of national efforts to monitor young people’s health. The survey receives funding through the national budget.

Norwegian municipalities initiate the survey themselves. All Norwegian municipalities are recommended to facilitate data collection every 3 years. Norwegian municipal authorities choose when and how they participate in the study. The most common and recommended recruitment approach is census sampling at the school level. There are variations based on the municipal composition each year, however, previous research has shown that Ungdata is nationally representative when analyzing a pooled sample over a period exceeding 3 years [27].

The survey consists of approximately 150 mandatory questions with the option of adding additional packages. Surveys are conducted throughout the school year from August to June and involve students attending Grades 8–10 and all 3 years of upper secondary school (generally between ages 13 and 19 years old).

In the current study, data that included questions about youth primary healthcare service utilization were used. These questions were included in the mandatory questionnaire from 2014 onwards. This produced five waves of data from 2014 to 2018. Response rates are reported to be high at the municipal level (> 80%). The sample for this study consisted of 46,019 participants (from 86 municipalities out of 422) in 2014, 73,426 (from 121 municipalities) in 2015, 70,577 (from 138 municipalities) in 2016, 107,601 (from 174 municipalities) in 2017 and 70,956 (from 124 municipalities) in 2018, resulting in a pooled sample of 368,579 young people from 416 (out of 422) municipalities in Norway. The gender distribution in our sample is 50% females and 50% males.

### Measures

Youth primary healthcare service utilization was measured by the question “How many times have you used the following healthcare services over the past 12 months?” Participants could choose from the following services: “school nurse or doctor”; “youth health centers”; “family doctor”; “psychologist”; “out-of-hours primary healthcare service”. The response options to indicate how often the participant used each service were as follows: “never” (1); “1–2 times” (2); “3–5 times” (3); “6 or more times” (4). For the analysis, these response categories were averaged to represent interval midpoint estimates of service use (never = 0, 1–2 times = 1.5, 3–5 times = 4, 6 or more times = 6).

Symptoms of psychological distress, sometimes referred to as psychological health complaints, were measured with a six-item scale derived from the widely used Hopkins Symptom Checklist (HSCL) [28]. The HSCL is recommended for use in both clinical and epidemiological studies to measure psychological distress among young people. Short formats of the HSCL (5–25 item scales) have been shown to perform almost as well as the full version [29]. Rasch analysis of the psychometric properties of the six-item HSCL scale used in the current study has previously shown that this scale works reasonably well [30]. Participants were asked if they had been affected by any of the following during the past week: “felt that everything is a struggle”, “had sleep problems”, “felt unhappy, sad or depressed”, “felt hopelessness about the future”, “felt stiff or tense”, “worried too much about things”. The six questions had four response options: (1) “not been affected at all”, (2) “not been affected much”, (3) “been affected quite a lot” and (4) “been affected a great deal”. To capture more severe psychological distress, the responses were dichotomized based on average scores greater than 3.0. Previous studies have shown that young people scoring above this cut-off point were within the range of depressive disorders commonly found in Norwegian community youth samples [31, 32].

The socioeconomic status of young people was measured using the Family Affluence Scale (FAS II) which is a validated, brief, assets-based measure of family wealth that is designed for use in youth surveys [33, 34]. FAS consist of four questions: “Does your family have a car?”; “Do you have your own bedroom?”; How many times have you travelled somewhere on holiday with your family over the past year?”; “How many computers does your family have?”. A mean score was created from the participants’ responses.

Availability of core primary healthcare services was measured through a municipal centrality index developed by Statistics Norway, ranking the 422 municipalities in Norway on a scale from 0 to 1000 based on service availability and available workplaces within a 90-min drive by car [35].

Physical health complaints are associated with psychological distress and is a predictor of health service utilization among young people, thus, we adjusted the regression analysis for the confounding effect of physical health complaints. Participants were asked “Have you had any of these health issues during the past month?”: “Headache”, “Neck and shoulder pain”, “joint and muscle pain”, “Stomach ache”, “Nausea/feeling sick” and “Palpitations”. Participants were then given the response options: “Never”, “A few times”, “Many times” and “Daily”. We dichotomized the responses based on one or more daily physical health complaint(s) during the last month. This approach is recommended by the Ungdata survey to capture more severe physical health complaints [6].

In addition, grade, gender and survey year (time) were added as covariates in later analysis.

### Data analysis

Statistical analysis was performed using the “survey” package for analysis of complex survey samples [36] in R [37]. Regression models were analyzed using a generalized linear model (GLM) with a log-link to account for non-normal distribution of the residuals. The GLM approach with a log-link is preferable to log-linearized models when the distribution of residuals is non-normal as was found in the current study [38, 39]. Complex survey design weights were created using population weights, stratifying for municipality and survey year, in order to maintain national representativeness of the sample (between-subject design). In addition, design weights were used to provide robust standard errors. The final regression model contains the following covariates: time (survey year), psychological distress, physical health complaints, grade, gender, FAS, availability, time:psychological distress (interaction term).

The interaction term between psychological distress and survey year were tested using a Rao-Scott log likelihood test for complex survey samples. The log likelihood statistic was used to test if an independent variable shows no association with the outcome (null hypothesis). If the likelihood test fails to reject the null hypothesis, removing the independent variable from the model will not substantially reduce the fit of that model [40].

## Results

### Descriptive statistics

Increasing trend of psychological distress and primary healthcare service utilization among young people between 2014 and 2018.

A summary of the population weighted trends in psychological distress between 2014 and 2018 suggests that high levels of psychological distress increased by 5.4% between 2014 and 2018, from 12.7% (95% CI = 12.1, 13.4%) in 2014, 13.6% (95% CI = 12.9, 14.3%) in 2015, 14.2% (95% CI = 13.5, 14.8%) in 2016, 16.7% (95% CI = 16.0, 17.5%) in 2017, to 18.1% (95% CI = 17.5, 18.7%) in 2018.

Overall, there were substantial gender and age differences in those suffering from psychological distress. For males, 7.5% (95% CI = 7.1, 8.0%) reported high levels of psychological distress compared to 22.5% among females (95% CI = 21.9, 23.1%). With respect to age, 21.9% (95% CI = 21.1, 22.8%) of the oldest youth (in the last year of upper secondary school) had high levels of psychological distress compared to 8.3% (95% CI = 7.9, 8.6%) among the youngest (in Grade 8).

In our sample, 34.7% (95% CI = 33.6, 35.8%) of the participants used the school health service (the school nurse or doctor), 12.8% (95% CI = 12.3, 13.3%) used a youth health center, 9.5% (95% CI = 9.3, 9.8%) used a psychologist, 60.1% (95% CI = 59.2, 60.9%) used their family doctor and 35.7% (95% CI = 35.3, 36.2%) used an out-of-hours primary healthcare service during the previous 12 months. In general, there was a trend of increasing service use in the youth population, increasing from an average of 3.6 consultations in 2014 to 4.1 consultations in 2018 (Table 1). This suggest that primary healthcare service utilization increased by 500 consultations per 1000 young people, between 2014 and 2018.

**Table 1:** The use of primary healthcare services among young people by year and psychological distress (population weighted)

Health service	Psychological distress	Mean number of consultations per youth (95% CI) Year					
		2014	2015	2016	2017	2018	Average, by distress
School nurse or doctor	High level	1.41 (1.34, 1.48)	1.38 (1.31, 1.45)	1.47 (1.38, 1.56)	1.48 (1.42, 1.54)	1.52 (1.46, 1.59)	1.46 (1.43, 1.49)
	Low level	0.62 (0.58, 0.67)	0.63 (0.59, 0.67)	0.64 (0.61, 0.67)	0.73 (0.69, 0.77)	0.79 (0.74, 0.85)	0.69 (0.66, 0.71)
Youth health centers	High level	0.72 (0.67, 0.76)	0.64 (0.58, 0.70)	0.63 (0.60, 0.67)	0.65 (0.61, 0.70)	0.65 (0.55, 0.75)	0.66 (0.63, 0.69)
	Low level	0.25 (0.22, 0.27)	0.21 (0.19, 0.23)	0.21 (0.20, 0.23)	0.26 (0.23, 0.28)	0.26 (0.24, 0.28)	0.24 (0.23, 0.25)
Psychologist	High level	1.22 (1.15, 1.28)	1.2 (1.16, 1.24)	1.19 (1.15, 1.24)	1.13 (1.1, 1.17)	1.11 (1.08, 1.14)	1.16 (1.14, 1.18)
	Low level	0.21 (0.20, 0.23)	0.22 (0.20, 0.23)	0.21 (0.20, 0.22)	0.25 (0.24, 0.25)	0.26 (0.24, 0.28)	0.23 (0.22, 0.24)
Family doctor	High level	2.20 (2.12, 2.28)	2.25 (2.20, 2.29)	2.24 (2.18, 2.30)	2.25 (2.15, 2.34)	2.22 (2.18, 2.26)	2.23 (2.20, 2.26)
	Low level	1.48 (1.44, 1.53)	1.51 (1.45, 1.56)	1.45 (1.42, 1.49)	1.53 (1.46, 1.59)	1.58 (1.48, 1.68)	1.51 (1.48, 1.55)
Out-of-hours primary healthcare service	High level	1.06 (0.99, 1.13)	1.12 (1.08, 1.17)	1.01 (0.97, 1.06)	1.02 (0.98, 1.05)	1.04 (1.00, 1.09)	1.05 (1.03, 1.07)
	Low level	0.70 (0.68, 0.72)	0.76 (0.73, 0.78)	0.70 (0.68, 0.73)	0.75 (0.74, 0.77)	0.80 (0.76, 0.83)	0.74 (0.73, 0.76)
**Average,** by year		3.64 (3.54, 3.74)	3.76 (3.62, 3.90)	3.64 (3.56, 3.72)	3.95 (3.86, 4.05)	4.13 (3.99, 4.27)	

Young people with high levels of psychological distress tended to consult primary healthcare services more often (M = 6.48, 95% CI = 6.40, 6.55), almost twice as much as their peers with low levels of distress (M = 3.36, 95% CI = 3.28, 3.43).

Looking more closely at the statutory youth primary healthcare services revealed that the use of the school health service increased from an average of 0.72 consultations per person in 2014 to 0.92 in 2018 (Table 1). Young people tended to consult youth health centers less often than the school health services. Consultation rates for youth health centers increased slightly from 0.31 per person in 2014 to 0.33 in 2018 (Table 1). Young people with high levels of psychological distress used school health services more than twice as much as their peers with low levels of distress (Table 1). The results were similar for youth health centers where young people with high levels of psychological distress used the service close to three times as much as their peers with low levels of distress (Table 1).

In general, there were gender and age differences in primary healthcare service use. Females and older users accessed the services more than males and younger users. On average, females had 1.5 more consultations during a 12-month period (M = 4.57, 95% CI = 4.50, 4.64) than males (M = 3.06, 95% CI = 2.96, 3.16). Older youth used primary healthcare services more than younger adolescents, with an average difference of 1.3 consultations between grade 8 (M = 3.30, 95% CI = 3.21, 4.30) and third year of upper secondary school (M = 4.58, 95% CI = 4.38, 4.72).

For the statutory youth services specifically, females tended to use both services twice as much as males. For the school health services, females had on average 1.07 consultations (95% CI = 1.04, 1.10) compared to 0.53 (95% CI = 0.51, 0.56) for males. For youth health centers, females had 0.42 consultations (96% CI = 0.40, 0.44) compared to 0.17 (95% CI = 0.17, 0.18) among males. As the young people went from lower to upper secondary school, they generally used the services more. For the school health service, use increased through grades 8 to 10, from 0.82 (95% CI = 0.78, 0.86) in grade 8 to a peak of 0.9 (95% CI = 0.87, 0.93) in grade 10. In the transition to upper secondary education where the availability of this service is reduced, the use of the school health service declined to 0.77 (95% CI = 0.74, 0.79). However, service use then increased to 0.8 (95% CI = 0.74, 0.86) in the third and final year of upper secondary school. For youth health centers, service use increased linearly from 0.17 (95% CI = 0.16, 0.18) in grade 8 to 0.51 (95% CI = 0.46, 0.56) in the last year of upper secondary school.

### Regression analysis

Convergence in primary healthcare service utilization for psychological distress among young people between 2014 and 2018.

Results from the GLM regression analysis suggest a changing pattern in primary healthcare service use among young people between 2014 and 2018. Results indicate a net change in service use over time. There was a significant yearly increase in all service types in the youth population (see Additional file 1). The statutory youth primary healthcare services, the school health service (school nurse or doctor) and youth health centers had an estimated yearly increase of 6 and 4% respectively, based on the log means of primary healthcare service use over time. For the remaining primary healthcare services, the use of a psychologist increased by 5% while both the use of family doctor and out-of-hours primary healthcare increased by 2% yearly.

Adding psychological distress as a covariate indicated the proportion of the effect of the time coefficient that could be explained by psychological distress among young people (Additional file 1). This ranged from 16% in the pattern of use for the school health service, 20% for out-of-hours primary healthcare services, 29% for family doctor, 56% for youth health centers, to 66% for the use of a psychologist. This suggests that increasing prevalence of psychological distress between 2014 and 2018 can explain a substantial part of overall primary healthcare service utilization among young people and that an indicator of psychological distress is important when explaining changes in primary healthcare service use over time. Furthermore, this may indicate that increasing prevalence of psychological distress in the youth population has a particularly strong effect on help seeking to youth health centers and psychologists.

The interaction term between psychological distress and time that was added to the model was significant and improved the model fit for the school health service (X^2^ (1, 635) = 8.9, *p* < 0.01), youth health centers (X^2^ (1, 635) = 8.6, *p* < 0.01), psychologist (X^2^ (1, 635) = 31.9, *p* < 0.001) and out-of-hours primary healthcare services (X^2^ (1, 635) = 14.5, *p* < 0.001), but not for family doctor (see Additional file 2). This indicates that primary healthcare service use follows a significantly different slope for young people depending on whether they have high levels of psychological distress or not. This effect did not change when adjusting for physical health complaints, gender, grade, socioeconomic status and service availability. The strongest predictor of primary healthcare service use was psychological distress followed by gender.

**Fig. 1:**
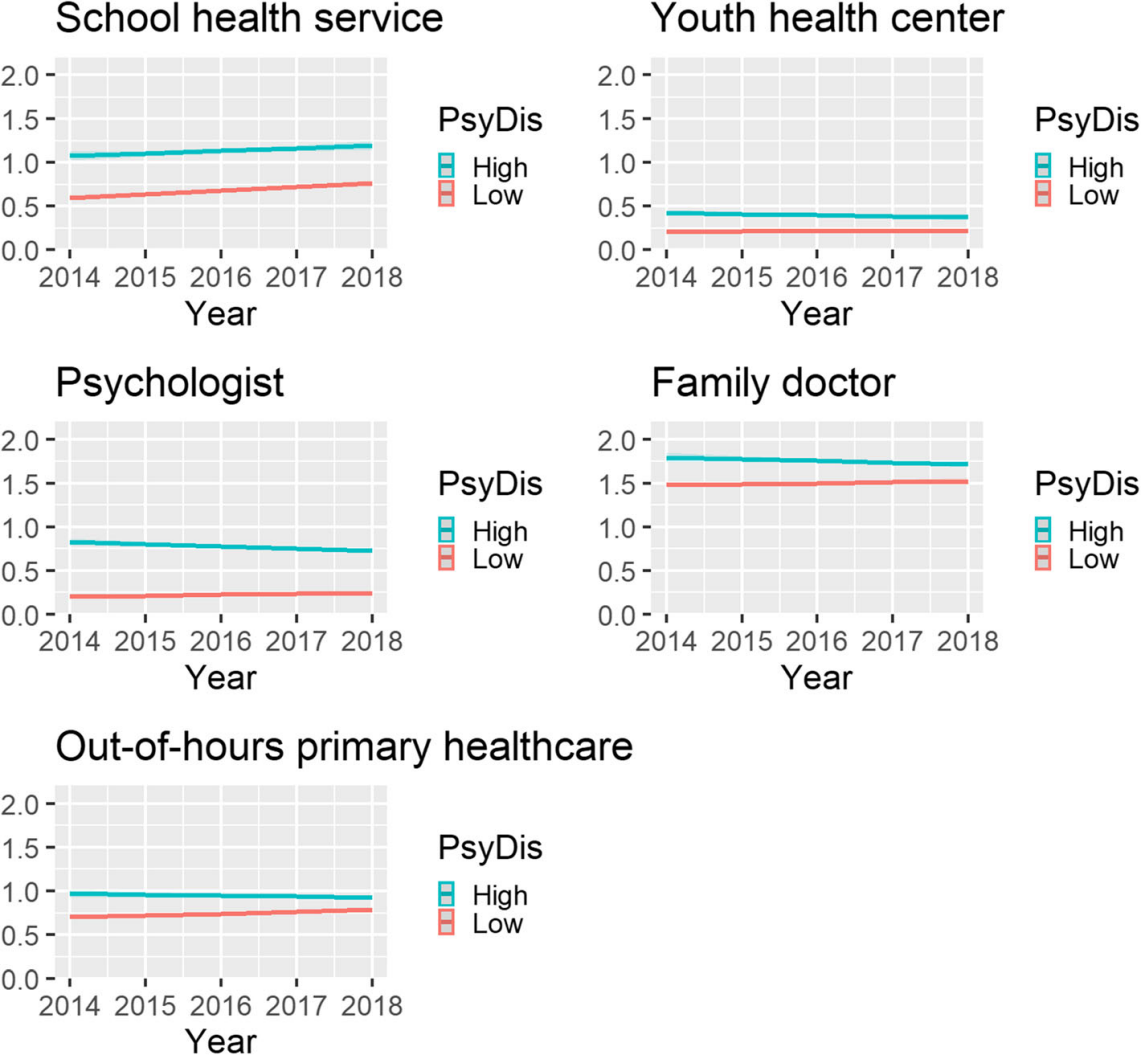
Youth primary healthcare service use by psychological distress and year (Mean number of consultations per youth)

Exponentiating the adjusted regression coefficients for primary healthcare service use over time at the average of all included covariates indicates that young people with high levels of psychological distress use health services more than their peers with low levels. However, the net increase of service use over time was mainly observed among young people with low levels of psychological distress and not among those with high levels (Fig. 1; see Additional file 2).

Based on the total predicted values, the observed increase in primary healthcare service use in 2014 compared to 2018 equated to an increase of 350 consultations per 1000 among young people with low levels of psychological distress, while it decreased by 150 consultations for young people with high levels of psychological distress. The only primary healthcare service which saw an actual increase in use by young people with high levels of psychological distress was the school health service (that is an increase of 120 consultations per 1000). This suggests convergence in primary healthcare service use between young people with low levels of psychological distress and those with high levels. If the current trend remains unchanged, young people with low levels of psychological distress will use primary healthcare services more than those with high levels within 10 years and the point of convergence is expected to be reached before the year 2029.

## Discussion

The aim of this study was to investigate the relation between youth primary healthcare service use and psychological distress in times of increasing mental health problems. Based on Andersen’s healthcare utilization model, we expected an increase in psychological distress, such as the increase seen among the youth of Norway, would lead to an increased need for primary healthcare services and an increase in help-seeking behavior among the distressed. We found that between 2014 and 2018 psychological distress among young people continued to increase alongside increasing rates of primary healthcare service use. A large proportion of young people used available primary healthcare services, ranging from 10% using a psychologist to 60% using their family doctor over a 12-month period. Overall, psychological distress has a conditional association with youth primary healthcare service use and can explain between 16 and 66% of the change in the use of services between 2014 and 2018, depending on the service type. Young people with high levels of psychological distress tended to seek help from primary healthcare services twice as often as their peers with low levels of distress. Contrary to our hypothesis, the absolute increase in primary healthcare service use observed between 2014 and 2018 appeared to be driven mainly by young people with low levels of psychological distress (350 net consultations more in 2018, per 1000) and not by the growing proportion of young people with high levels of psychological distress (150 net consultations *less* in 2018, per 1000). While young people with low psychological distress use services more over time, young people with high levels of psychological distress use services less and less. This is suggestive of a converging trend. This decrease in service utilization among distressed young people was seen in all the youth primary healthcare services, except in the school health service.

Why do young people with high levels of psychological distress use primary healthcare services less over time despite the increasing prevalence of psychological distress?

In our data, based on self-reports, the proportion of young people with high levels of psychological distress increased from 13% in 2014 to 18% in 2018. An increase in psychological distress in Norway has previously been observed by other researchers in the period between 1992 and 2018 [4–8]. This increasing trend of mental health problems among young people is regarded as a public health concern [4]. Psychological distress is usually considered to be strongly associated with primary healthcare service utilization among young people [12, 13] and according to Andersen’s health service utilization model [25], increasing rates of psychological distress should theoretically lead to an increased need for primary healthcare services. For young people this should constitute increased primary healthcare service utilization within the school health service and youth health centers or other primary healthcare services. Superficially, this appeared to be the case as we found that young people with psychological distress tend to use primary healthcare services twice as much as their peers with low levels of distress. However, between 2014 and 2018, youth service utilization of primary healthcare services declined yearly by 150 consultations per 1000 young people among those with high levels of psychological distress.

The cause of the decline in the utilization of primary healthcare services among distressed young people is unknown and was unexpected based on our proposed theoretical framework. The healthcare utilization model suggests that deteriorating health outcomes would create a greater need for care leading to greater primary healthcare service utilization. However, since our study does not support this idea, the implication is that there may be serious barriers between perceived need for care by young people and primary healthcare service access. This is in line with previous research that has shown that young people access services less than adults [17]. The possible barriers between care needs and help-seeking behavior is particularly worrying in times of the increasing prevalence of psychological distress in the youth population.

At the present, it is not clear whether young people with high levels of psychological distress seek help elsewhere within the healthcare system (for example within the specialized healthcare service or from other care services), from informal sources (such as family and friends) or whether their healthcare needs remain unmet. There are indications that the proportion diagnosed with a mental health related disorders in the specialist health service (BUP) increased between 2011 and 2016, from 5 to 7% per year (mainly depression, anxiety-, adjustment- and eating disorder) among adolescent girls. The trend remained stable among younger children and boys. It is feasible that young people with high levels of psychological distress (especially adolescent girls) are more frequently referred from primary healthcare to the specialist healthcare in 2018 compared to 2014. However, according to the Norwegian Institute of Public Health the cause of increased referrals to the specialist healthcare service among adolescent girls remains unknown, although it is considered unlikely that direct changes in referral or diagnostic practices has contributed to the increase due to the fact that there have been no changes in other age groups [24]. Therefore, since primary healthcare services still function as a “gatekeeper” for referrals to the specialized healthcare service and since the prevalence of psychological distress appears to be increasing, one would still expect an increase of youth help seeking from primary healthcare services, contrary to the findings in the current study.

Other researchers highlight the paradoxical nature of increased provision of healthcare services, expenditure and utilization combined with a lack of improvements in mental health outcomes - one would expect that when healthcare services are effective, psychological distress and mental health outcomes would be improved [41]. Thus, it is the responsibility of the healthcare system to provide equitable primary healthcare proportionate to healthcare needs in order to reduce psychological distress among young people. Great measures are taken in Norway to ensure high quality, easily available youth primary healthcare, but the extent to which the services provided are standardized and evidence-based is still under debate. Several Norwegian systematic reviews suggest that there is still room for improvement in the provision of youth primary healthcare services [42, 43]. The authors argue that the lack of improved mental health may be related to the quality of the care service provided and that currently these services do not meet the minimum standards of clinical practice guidelines. In addition, the services may fail to adequately provide preventive efforts in the pursuit of health promotion among young people and adults. This may suggest that the quality of the services provided for young people in Norway does not currently meet the needs of young people with psychological distress and that many distressed young people seek help elsewhere or possibly not at all.

Our finding that young people with high levels of psychological distress used primary healthcare services less between 2014 and 2018, despite increased prevalence of psychological distress, supports the recommended quality improvements in healthcare systems suggested by Jorm et al. [41], in order to make primary healthcare services more effective in preventing further deterioration of mental health in the youth population and in order to ensure equitable primary healthcare service utilization proportionate to healthcare need.

Concerningly, at the present time there is no known explanation as to why young people with high levels of psychological distress utilize primary healthcare services less over time despite the growing proportion of psychological distressed youth in the Norwegian population. Policy makers and care providers should be advised that primary healthcare services should be made more attractive and youth-friendly in order to ensure equitable access for young people with high levels of psychological distress.

### Why do young people with low levels of psychological distress use services more often over time?

Young people with low levels of psychological distress use primary healthcare services relatively less than those with high levels of psychological distress. However, between 2014 and 2018, service use among young people with low levels of psychological distress increased substantially. Compared to 2014, young people with low levels of psychological distress had 350 more primary healthcare consultations per 1000 young people in 2018. This leads to many questions regarding psychological distress and its effects on primary healthcare utilization. Since young people with low levels of psychological distress use primary healthcare services more often over time, this may suggest a change in the propensity to seek care, such as a lower threshold for seeking healthcare [44, 45]. Generally, it is agreed upon that utilization of primary healthcare services among young people is low, so a general increase in the use of primary healthcare services among the less distressed could be viewed as something positive. However, “overuse” occurs when the threshold for seeking care is lower than the expected healthcare need [27].

A previous study found that the mean level of psychological distress among young people using psychiatric services between 2002 and 2010 decreased suggesting a lower threshold for help-seeking behavior among young people in Sweden. However, this development was not found among adults. The authors therefore argued that a lower threshold of help-seeking behavior among young people may explain the recent increase in psychiatric service use in Sweden and perhaps in other developed countries [13]. Another study involving patient data from 34 countries investigated the propensity to seek healthcare and found that it was weakly associated with greater use of a family doctor [46]. In addition, the authors found that the characteristics of the healthcare system might influence patients’ decision to seek help, potentially leading to either overuse or underuse of health services [46]. According to the Norwegian Institute of Public Health [44], underuse of primary healthcare services appears to be more likely in the general population. On the other hand, the Norwegian Institute of Public Health points out that if help-seeking behavior were to increase among those with low levels of psychological symptoms as opposed to those with high levels of symptoms, as seen in our study and in a study by Kosidou et al. [13], this might instead indicate overuse of health services.

Our finding of increased primary healthcare service utilization among the less distressed are rather unexpected, based on the healthcare utilization theory, and it is a topic that has seldomly been explored in research. Subsequently, it is unknown to what extent this may represent a general phenomenon, reflecting greater societal awareness- or reduced stigma of mental health problems [13, 45]. Other researchers, however, suggest a more specific cause of increased primary healthcare utilization, specific to Norwegian youth. Bakken et al. [47] suggest that a school-absence policy to reduce truancy, introduced in Norway in 2016, is likely to have caused inflated healthcare utilization rates of family doctors by young people, not related to morbidity. From the start of the 2016/17 school year, new rules for absence were introduced in upper secondary schools [48]. The main feature of the new regulations is that students with more than 10% undocumented absence in a school subject lose their right to a graded semester assessment without which they are unable to graduate. In case of illness, only a medical certificate or documentation issued by a qualified healthcare professional will be considered as valid absence documentation following more than 10% absence from upper secondary school. According to Bakken et al. [47], the number of consultations in the general practice services increased by 30% in the age group 16 to 18 years in the year the reform was introduced compared to the previous year. This led the authors to believe that it is very likely that the school absence policy is inadvertently causing young people to overuse primary healthcare services. Bakken et al. [47] only included data on the use of family doctors among young people, but since health personnel (e.g., physiotherapists, dentists, psychologists) other than the family doctor may also document absence for the students [48], a similar increase could conceivably be observed in other primary healthcare services as well. Interestingly, our study partially confirms this. Young people in general (and to a greater extent young people in upper secondary school) with low levels of psychological distress had a general increase in primary healthcare service use after the policy change in 2016 above that of young people with high levels of psychological distress. This might suggest that part of the increase in youth primary healthcare service use is not a sign of increased morbidity, but rather in part due to young people needing a medical certificate to avoid failing subjects, further supporting the notion of overuse among young people with low levels of psychological distress.

Alternatively, the increased use of primary healthcare services among young people with low levels of psychological distress might partly be explained by a change in how young people utilize primary healthcare services for matters not directly relating to psychological distress and not captured in our study. Young people use primary healthcare services for a wide range of reasons, including sexual health (such as pregnancy, contraception and sexually transmitted infections) which is also one of the most common reasons for contact with youth primary healthcare alongside mental health issues [16].

It is challenging to elucidate on the cause of increasing primary healthcare utilization among young people with low levels of psychological distress. On the one hand, young people are known to utilize healthcare services less than adults, therefore a lowered threshold to seek-help may be seen as positive. On the other hand, since the trend of utilization of primary healthcare is disproportionate to expected healthcare needs this might also represent inequities in youth primary healthcare service utilization among young people. In addition, indications of inflated utilization rates not related to morbidity among the less distress youth are a worrying development that warrant further investigation. The propensity for young people to seek care remains understudied and more research is needed in order to determine if increased utilization of primary healthcare services among young people with low levels of psychological distress are due to overuse mechanisms.

### Predisposing and enabling factors of service use

The predisposing and enabling factors covered in this study, gender, age, socioeconomic status, service availability and psychological distress, are all important indicators of primary healthcare service utilization among young people. In regard to gender and youth primary healthcare service use, it is well known that females use primary healthcare services more than males, a finding that is also observed in the current study. Given that males have the same need for primary healthcare services when they have psychological distress, it is not thoroughly understood why males report less help-seeking behavior. Empirical evidence indicates that low treatment rates among males cannot be explained by better health but must be attributed to a discrepancy between perception of need and help-seeking behavior [49, 50]. Furthermore, we found that older young people generally used primary healthcare services more often.

In terms of service availability, we found that youth primary healthcare utilization generally increased when services were more widely available.

We also found that overall, socioeconomically disadvantaged young people used primary healthcare services more than the more advantaged. This might suggest services utilization proportionate to those with the greatest need [51].

### Strengths and limitations

A major strength of this study is that it is based on a very large sample of young people and has a yearly data collection scheme allowing the investigation of trends in the general youth population in Norway. In addition, data are weighted and adjusted in order to ensure national representativeness of young people growing up in Norway [27]. Sensitivity analysis of descriptive unweighted data and the weighted estimates show only minor differences between these estimates, further indicating that there are no systematic differences between different survey waves at the population level of analysis. However, one limitation is that the repeated cross-sectional design used in the current study does not enable strict causal inference. In addition, this study relies on self-reported data, which provide the overall picture from the young people’s own perspective and might be distinct from objective data. Moreover, subgroup analysis of primary healthcare services disparities in vulnerable groups could inform this research. However, such data is not available due to ethical guidelines in Norway discouraging studying certain participant characteristics. Finally, the use of youth primary healthcare services is a general outcome in the survey and therefore the specific reason for the consultations are not known and could be related to health outcomes other than those studied in our paper.

## Conclusion

Between 2014 and 2018, there was an increase in the prevalence of psychological distress and primary healthcare service use among young people in Norway. Young people with high levels of psychological distress used primary healthcare services twice as often as their peers. However, despite an increasing proportion of young people with psychological distress between 2014 and 2018, primary healthcare service use among young people with high levels of psychological distress declined. This suggests that the absolute increase seen in primary healthcare utilization among young people is accounted for by increased use among the less distressed. Declining primary healthcare service use among young people with high levels of psychological distress in parallel with increasing use among the less distressed suggests future convergence. A converging trend of primary healthcare service use may suggest overuse of primary healthcare services among the less distressed young people and likely underuse among the more distressed. It is currently unknown if young people with high levels of psychological distress seek care elsewhere in the healthcare system, from friends and family or not at all. These issues need to be further investigated in order to map possible overuse and underuse mechanisms and to ensure that young people get excellent care when they need it.

## Supplementary Information

**Additional file 1.** Changes in the (log) year coefficient for primary healthcare services use among young people.

**Additional file 2.** Generalized linear regression of primary healthcare services use by young people 2014–2018.

## Data Availability

The datasets generated during and/or analyzed during the current study are available in the NSD - Norwegian Centre for Research Data repository (https://nsd.no/nsd/english/orderform.html).

## Abbreviations

BUP: The Division of Children and Adolescents’ Psychiatric Polyclinic Services
FAS: Family Affluence Scale
GLM: Generalized Linear Model
HSCL: Hopkins Symptom Checklist
NOVA: Norwegian Social Research – NOVA
NSD: Norwegian Centre for Research Data
