# Supplementary material for: Trends in the utilization of youth primary healthcare services and psychological distress
Source: BMC Health Serv Res. 2021 Feb 3;21:115. doi: 10.1186/s12913-021-06124-w (PMC7860003; doi:10.1186/s12913-021-06124-w)
Supplement: Supplementary file 1 — Additional file 1. Changes in the (log) year coefficient for primary healthcare services use among young people. [file 12913_2021_6124_MOESM1_ESM.docx]

Additional file 1. Changes in the (log) year coefficient for primary healthcare services use among young people.

| Model | **School nurse or doctor** | **Youth health centres** | **Psychologist** | **Family doctor** | **Out-of-hours primary healthcare service** |
| --- | --- | --- | --- | --- | --- |
| A: Time | 0.06 | 0.04 | 0.05 | 0.02 | 0.02 |
| B: Time and psychological distress | 0.05 | 0.02 | 0.02 | 0.01 | 0.02 |
| % of time effect accounted for | 16 | 56 | 66 | 29 | 20 |
| Model fit | X^2^ (1, 636) = 2492.2, p < 0.001 | X^2^ (1, 636) = 4459.8, p < 0.01 | X^2^ (1, 636) = 5833.8, p < 0.001 | X^2^ (1, 636) = 3793.5, p < 0.001 | X^2^ (1, 636) = 3512.1, p < 0.001 |
