# Supplementary material for: Trends in the utilization of youth primary healthcare services and psychological distress
Source: BMC Health Serv Res. 2021 Feb 3;21:115. doi: 10.1186/s12913-021-06124-w (PMC7860003; doi:10.1186/s12913-021-06124-w)
Supplement: Supplementary file 2 — Additional file 2. Generalized linear regression of primary healthcare services use by young people 2014–2018. [file 12913_2021_6124_MOESM2_ESM.docx]

Additional file 2. Generalized linear regression of primary healthcare services use by young people 2014-2018.

|  | **School health service** | | | **Youth health centers** | | | **Psychologist** | | | **Family doctor** | | | **Out-of-hours primary healthcare services** | | |
| --- | --- | --- | --- | --- | --- | --- | --- | --- | --- | --- | --- | --- | --- | --- | --- |
| **Predictors** | **Log-Mean** | **95 % CI** | **p** | **Log-Mean** | **95 % CI** | **p** | **Log-Mean** | **95 % CI** | **p** | **Log-Mean** | **95 % CI** | **p** | **Log-Mean** | **95 % CI** | **p** |
| (Intercept) | -0.697 | -0.794 – -0.601 | <0.001 | -2.127 | -2.319 – -1.934 | <0.001 | -1.234 | -1.357 – -1.111 | <0.001 | -0.399 | -0.456 – -0.343 | <0.001 | -0.915 | -0.984 – -0.846 | <0.001 |
| Time | 0.062 | 0.042 – 0.082 | <0.001 | 0.014 | -0.017 – 0.044 | 0.377 | 0.048 | 0.029 – 0.067 | <0.001 | 0.007 | -0.002 – 0.016 | 0.143 | 0.029 | 0.013 – 0.044 | <0.001 |
| Psychological distress | 0.588 | 0.534 – 0.643 | <0.001 | 0.71 | 0.624 – 0.796 | <0.001 | 1.413 | 1.352 – 1.474 | <0.001 | 0.194 | 0.158 – 0.230 | <0.001 | 0.327 | 0.277 – 0.377 | <0.001 |
| Physical health complaints | 0.299 | 0.285 – 0.313 | <0.001 | 0.43 | 0.397 – 0.462 | <0.001 | 0.602 | 0.573 – 0.632 | <0.001 | 0.415 | 0.399 – 0.430 | <0.001 | 0.467 | 0.447 – 0.486 | <0.001 |
| Grade | -0.045 | -0.056 – -0.033 | <0.001 | 0.216 | 0.194 – 0.237 | <0.001 | 0.067 | 0.057 – 0.076 | <0.001 | 0.093 | 0.087 – 0.099 | <0.001 | -0.036 | -0.045 – -0.028 | <0.001 |
| Gender | 0.53 | 0.505 – 0.555 | <0.001 | 0.655 | 0.602 – 0.709 | <0.001 | 0.387 | 0.354 – 0.420 | <0.001 | 0.161 | 0.144 – 0.178 | <0.001 | -0.097 | -0.122 – -0.071 | <0.001 |
| FAS | -0.093 | -0.113 – -0.073 | <0.001 | -0.061 | -0.099 – -0.022 | 0.002 | -0.349 | -0.388 – -0.310 | <0.001 | 0.114 | 0.102 – 0.127 | <0.001 | 0.227 | 0.209 – 0.244 | <0.001 |
| Availability | 0.001 | 0.001 – 0.001 | <0.001 | 0.001 | -0.001 – 0.001 | 0.339 | 0.001 | 0.001 – 0.001 | <0.001 | 0.001 | 0.001 – 0.001 | <0.001 | 0.001 | -0.001 – 0.001 | 0.755 |
| Time:Psychological distress | -0.036 | -0.063 – -0.009 | 0.01 | -0.046 | -0.079 – -0.013 | 0.007 | -0.081 | -0.107 – -0.055 | <0.001 | -0.018 | -0.035 – -0.001 | 0.033 | -0.04 | -0.061 – -0.019 | <0.001 |
